# Hypermethylation of the Promoter of miR-338-5p Mediates Aberrant Expression of ETS-1 and Is Correlated With Disease Severity Of Astrocytoma Patients

**DOI:** 10.3389/fonc.2021.773644

**Published:** 2021-11-11

**Authors:** Junping Wang, Cheng Huo, Jinzhu Yin, Lixia Tian, Lili Ma, Dongsheng Wang

**Affiliations:** ^1^ Department of Neurosurgery, The Second Affiliated Hospital of Dalian Medical University, Dalian, China; ^2^ Department of Neurosurgery, The Sinopharm Tongmei General Hospital, Datong, China; ^3^ Department of Neurology, The Yantaishan Hospital, Yantai, China

**Keywords:** noncoding RNAs, microRNA-338-5p, E26 transformation-specific sequence 1, DNA methylation, astrocytoma, DNA methyltransferase 1

## Abstract

The pro-oncogene ETS-1 (E26 transformation-specific sequence 1) is a key regulator of the proliferation and invasion of cancer cells. The present work examined the correlation of the aberrant expression of ETS-1 with histological or clinical classification of astrocytoma: grade I (pilocytic astrocytoma), grade II (diffuse astrocytoma), grade III (anaplastic astrocytoma), and grade IV (glioblastoma multiforme). MicroRNA, miR-338-5p, was predicted by an online tool (miRDB) to potentially target the 3’ untranslated region of ETS-1; this was confirmed by multi-assays, including western blot experiments or the point mutation of the targeting sites of miR-338-5p in ETS-1’s 3’untralation region (3’UTR). The expression of miR-338-5p was negatively associated with that of ETS-1 in astrocytoma, and deficiency of miR-338-5p would mediate aberrant expression of ETS-1 in astrocytoma. Mechanistically, hypermethylation of miR-338-5p by DNA methyltransferase 1 (DNMT1) resulted in repression of miR-338-5p expression and the aberrant expression of ETS-1. Knockdown or deactivation of DNMT1 decreased the methylation rate of the miR-338-5p promoter, increased the expression of miR-338-5p, and repressed the expression of ETS-1 in astrocytoma cell lines U251 and U87. These results indicate that hypermethylation of the miR-338-5p promoter by DNMT1 mediates the aberrant expression of ETS-1 related to disease severity of patients with astrocytoma.

## Introduction

Astrocytoma, which originates from astrocytes in the brain, is the most common and fatal type of brain tumor (glioma) (1–5). There are four histological or clinical grades of the disease: grade I (pilocytic astrocytoma) (6), grade II (diffuse astrocytoma) (7), grade III (anaplastic astrocytoma) (8), and grade IV (glioblastoma multiforme, GBM) (9). More severe astrocytoma (i.e., grades III and IV) (10–12) is associated with worse prognosis and more aggressive disease. Accumulating evidence indicates that grade I astrocytomas can be considered to be benign and are associated with long-term survival. Patients with grade II astrocytoma also have a survival of approximately 7–8 years (13–15). However, patients with grade III astrocytoma have an average/median 2–3 year survival, and the median survival of patients with GBM (grade IV astrocytoma) is only 9–14 months (13–15). Detection of protein factors related to the occurrence and progression of the disease in clinical specimens of different grades is helpful for diagnosis, and can also help to clarify the molecular mechanisms underlying disease progression.

The transcription factor ETS-1 (E26 transformation-specific sequence 1) is an important regulator of malignant tumor cell proliferation that also has central roles in metastasis and invasion (16–18). Some evidence indicates that ETS-1 is closely related to the progression of a variety of tumors, including breast cancer and liver cancer, and its high expression is positively correlated with the degree of tumor malignancy (19, 20). MicroRNAs (miRs) are a type of small non-coding RNA (generally 20–25 nucleotides in length) transcribed and synthesized by RNA polymerase II (21, 22). In cells, miRs can act as tumor suppressors by directly acting on the 3’ untranslated regions (3’UTRs) of pro-oncogenes to inhibit their expression (23–25). The deficiency of miRs as tumor suppressors, represented by miR-34a, are considered an important mechanism of cell carcinogenesis (26–28). Promoter methylation is considered to be a promising mechanism for the deficiency of miRs as tumor suppressors (28–30). In the present work, the expression of ETS-1 and miR-338-5p was detected in a series of clinical specimens. The results revealed that the DNMT1-mediated hypermethylation of the miR-338-5p promoter region could participate in the loss of miR-338-5p expression and the high expression of ETS1 associated with disease severity in astrocytoma patients.

## Materials and Methods

### Clinical Specimens and Cell Lines

The collection and use of clinical specimens and the protocol of the present work were all reviewed and approved by the Second Affiliated Hospital of Dalian Medical University. Written consent was obtained from all patients. The pathological grades of tumor tissues were confirmed according to the World Health Organization histological classification. Thirty specimens each of grade of astrocytoma (grades I–IV) were included in the present work. All the experiments using human-related materials were performed according to the principles described in the Declaration of Helsinki (1986).

### The Small Molecular Inhibitors Used in the Presence Work

The small molecular inhibitors used in the presence work, Decitabine (the Cat. No.: S1200), Azacitidine (the Cat. No.: S1782) or Trichostatin A (TSA, the Cat. No.: 1045) was purchased from the Selleck Corporation, Houston, Texas, USA. The pure powders of these drugs (purity greater than 98.7%) used in this study was dissolved by the dimethyl sulfoxide (DMSO) and diluted by the DMEM without FBS (17, 31, 32). The solution of the agents was filtered using a sterile 0.22μm pore filter membrane.

### The Lentivirus Particles

Contains wild-type full-length ETS-1 (that is, contains 3’UTR) sequence or mutant (mutation of miR-338-5p action site in ETS-1 3’UTR) full-length ETS-1 (that is, contains 3’UTR) sequence, hsa-pre-miR-338, siDNMT-1 (containing siRNA sequence of DNMT-1 in pSilencer 2.1 U6 vector) sequence. The cells were transected by using these vectors by using the G418 (33–35). 

### Bisulfite PCR and Next-Generation Sequencing (NGS) Methods

BSP-NGS was performed according to the methods described by Ma et al. (2020) and He et al. (2021) (28, 30). Briefly, genomic DNA samples were extracted from clinical tissues or cultured cells using a DNeasy Blood & Tissue Kit (Cat No. 69504; QIAGEN Corporation, Hilden, Nordrhein-Westfalen, Germany), then treated with an EpiTect Bisulfite Kit (Cat No. 59104; QIAGEN) according to the manufacturer’s instructions. BSP was performed using Platinum II Hot-Start PCR Master Mix (Cat No.: 14000012, Thermo Fisher Scientific, Waltham, Massachusetts, USA) to amplify the promoter region of miR-338-5p with CpG sites/islands. The BSP products were directly sequenced using Ion Torrent PGM (Cat. No. 4462921; Thermo Fisher Scientific), an Ion PGM HI-Q View Sequencing 200 kit, and Torrent Suite 5.6 and Ion Reporter 5.6 software (Life-technology, Thermo Fisher Scientific). The 2000-base-pair (bp) sequence upstream of miR-338-5p (the promoter region of has-pre-miR-338) from the human genome (GRCh38/hg38) was obtained from the UCSC database (http://genome.ucsc.edu/index.html). Methyl Primer Express v. 1.0 was used to predict the CpG islands/sites located in the promoter region of miR-338, and to design a BSP primer for amplification (amplification length: 259 bp). The primer sequence was as follows: forward primer, 5’-GYGTATGGTTTGTGAGGT-3’ (Y = C/T, mixed primer); reverse primer, 5’-TAAATATCAAACCATTATCTTCCC-3’.

### Prediction of miRs Potentially Targeting EST-1, and The Cancer Genome Atlas (TCGA)

The miRs potentially targeting ETS-1 were predicted using the online tool miRDB, and those with high scores were selected as candidates. The expression levels of miRs in para-cancerous non-tumor tissues were obtained from the GEPIA database, TCGA, and the GTEx project using a standard processing pipeline (36, 37). The sequences of the miRs or the ETS-1 with its 3’UTR were searched in NCBI to support the prediction results from online tool miRDB and the interaction between miR-338-5p with ETS-1 was further confirmed by using multi-assays. The endogenous expression levels of miR-338-5p, DNMt-1, ETS-1, and related factors in astrocytoma specimens were examined by quantitative PCR (qPCR) according to the methods described by Chi et al., Yang et al., and Meng et al. (38–40). The CT values of the target genes (such as ETS-1 or hsa-pre-miR-338-5p, etc. and the internal reference β-Actin) obtained by qPCR detection are calculated, and the expression levels of these target genes are finally calculated (ie folds to β- Actin). Thereafter, the expression level of the target gene in any specimen is converted to “1” for conversion, and finally the relative expression level of the specific gene in all specimens is obtained. The following primers were used: (1) has-pre-miR-338, stem–loop primer 5’-GTCGTATCCAGTGCGTGT CGTGGAGTCGGCAATTGCACTGGATACGACCACTCAG-3’, forward primer 5’-CCTGGTGCTGAGT GGTCG-3’, reverse primer 5’-CAGTGCGTGTCGTGGAGT-3’; (2) ETS1, forward primer 5’-GAGTCAACCCAGCCTATCCAGA-3, reverse primer 5’-GAGCGTCTGATAGGACTCTGTG-3’; (3) DNMT-1, forward primer 5’-AGGTGGAGAGTTATGACGAGGC-3’; reverse primer 5’-GGTAGAATGCCTGATGGTCTGC-3’; (4) MMP-3, forward primer 5’-CACTCACAGA CCTGACTCGGTT-3’, reverse primer 5’-AAGCAGGAT CACAGTTGGCTGG-3’; (5) MMP-9, forward primer 5’-GCCACTACTGTGCCTTTGAGTC-3’, reverse primer, 5’-CCCTCAGAGAATCGCC AGTACT-3’; (6) MMP-1, forward primer 5’-CCAAATGGGCTTGAAGCTG-3’, reverse primer 5’-GGTATCCGTGTAG CACATTCTFTC-3’; (7) GAPDH, forward primer 5’-ACATCAAGAAGGTGGTGAAGCAGG-3’, reverse primer 5’-AGCTTGACAAAGTGGTCGTTGAGG-3’.

### Western Blotting

Two astrocytoma cell lines, U251 and U87, were purchased from the National Infrastructure of Cell Line Resources of Chinese Government, Chinese Academy of Medical Sciences/Peking Union Medical University, Beijing, China. Antibodies against the ETS1 (cat. no. ab220361) and DNMT-1 (cat. no. ab188453) were purchased from Abcam Corporation (Cambridge, UK). The antibody against the intracellular domain of Notch protein was a gift from Dr. Yingshi Zhang of Shenyang Pharmaceutical University, Shenyang City, Liaoning Province, China. The cells were cultured and protein samples were extracted and analyzed using standard western blotting methods (41–43). GAPDH (cat. no. ab8245) was used as the loading control. Images of protein bands were quantitatively analyzed using Image J software (National Institutes of Health, Bethesda, MD, USA) as described in a previous publication (28). Use Image J software to quantitatively analyze images of images from Western blot, the specific method is: Use Image J software to mark the bands of proteins, and determine the amount of protein expression level based on the total intensity of bands (the area of the bands and grayscale-value intensity). The expression level of ETS-1 or DNMT-1 was corrected by the loading control (GAPDH) (folds of GAPDH). Take the control group as “1” to measure the relative protein expression values of each group.

### Cell Culture and Transwell Experiments

U87 or U251 cells were cultured in Dulbecco’s modified Eagle medium (DMEM) containing 10% fetal bovine serum (FBS) and transfected with plasmids. Then, cells were harvested for *in vitro* invasion (transwell) experiments (38). Extracellular matrix (Sigma-Aldrich, Merck & Co., Inc., Kenilworth, New Jersey, USA; diluted 1:10 with DMEM without FBS) was pre-added to the chambers of the transwell plates. Then, the cells were harvested and prepared as single-cell suspension *via* DMEM; and the cell suspensions of U87 or U251 cells were added to the inner chambers (20000 cells per chamber). Next, the transwell plates were incubated at 37°C in 5% CO_2_ for 12 h. The transwell plates were mixed with absolute ethanol and stained with purple-crystal (0.5%, w/v). Photographs of invading cells were taken and quantitatively analyzed with Image J (USA National Institutes of health [NIH], Bethesda, Maryland, U.S.) (28). Use Image J software to quantitatively analyze Invaded cells, the specific method is: Use Image J software to mark the cells, and determine the amount of Invaded cells based on the total number of pixels in the photo and the number of circled Invaded cells. Take the amount of invaded cells in the control group as “1” to determine the relative number of Invaded cells in each group.

### Subcutaneous Tumor Model

The animal experiments included in the present work were performed in accordance with the UK Animals (Scientific Procedures) Act 1986 and the associated guidelines. U87 or the U251 cells were cultured and transfected with vectors according to the manufacturer’s instruction (the Vigene Corporation, Jinan City, Shandong Province, China). Then, the cells were harvested and cell suspensions were injected subcutaneously (near the veins located in the groin on the inside of the lower extremities) into the nude mice (44). At the injection, mice were reared for 4–8 weeks; then, the mice were sacrificed and tumor tissues were collected for calculation of tumor volumes and weights. Tumor volumes were calculated using the following formula: tumor width × tumor width × tumor length/2. Tumor weights (g) were determined using a high-precision electronic balance.

### Statistical Analysis

Statistical analysis was performed by SPSS statistical software (software version 9.0, the IBM Corporation, Armonk, NY, the USA). A P value < 0.05 was considered as the significant difference between two groups.

## Results

### Endogenous Expression of ETS-1 Is Associated With Disease Severity of Astrocytoma

**Figure 1:**
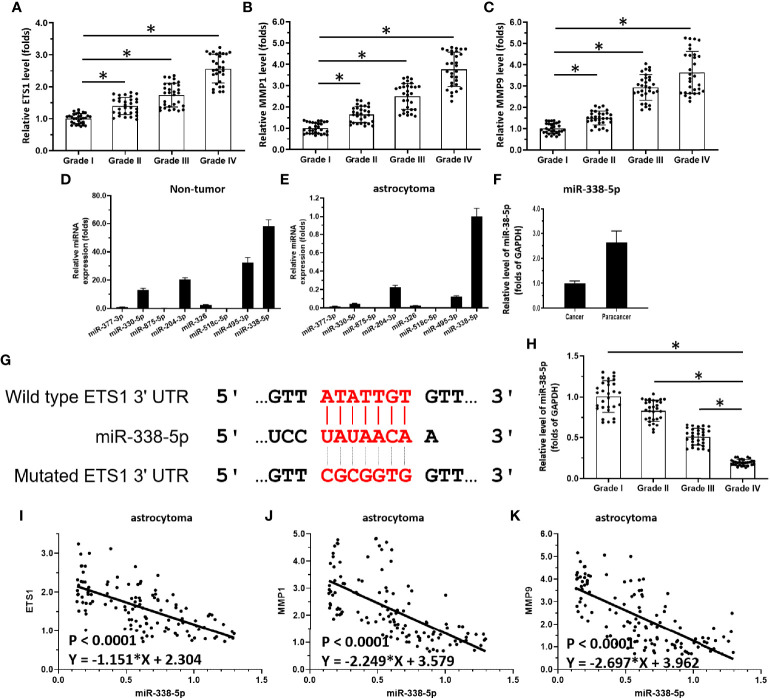
miR-338-5p/ETS-1 is correlated with disease severity in human astrocytoma patients. **(A–C)** Scatter histograms showing expression levels of ETS-1 **(A)**, MMP1 **(B)**, and MMP9 **(C)** in grade I–IV astrocytoma as determined by qPCR. **(D, E)** Expression levels of miRs potentially targeting ETS-1 in para-tumor/non-tumor tissues or astrocytoma tumor tissues. **(F)** Comparison of miR-338-5p levels in para-tumor/non-tumor tissues and astrocytoma tumor tissues. **(G)** Sequence of ETS-1 and its interaction with miR-338-5p. **(H)** Scatter histogram showing expression levels of miR-338-5p in grade I–IV astrocytoma as determined by qPCR. **(I–K)** Scatter plots showing correlations of miR-338-5p levels with those of ETS-1 **(I)**, MMP1 **(J)**, and MMP9 **(K)**. The statistical analysis was performed by student T-test without two-tails **(A-C, H)** and linear regression **(I-K)** by SPSS statistical software. *P < 0.05.

First, the endogenous expression of ETS-1-related factors in astrocytoma was examined. As shown in **Figure 1A**, the expression level of ETS-1 in clinical specimens gradually increased from grade I to grade IV astrocytoma, with the highest expression level in grade IV. MMPs (matrix metalloproteinases), an invasion-related downstream gene of ETS-1, showed a similar expression trend to that of ETS-1 in astrocytoma (**Figures 1B, C**). These results show that the aberrant activation of the ETS-1 pathway is correlated with the severity of astrocytoma.

### Deficiency of miR-338-5p Participates in the Aberrant Activation of ETS-1 in Astrocytoma

To explore potential mechanisms of aberrant activation of the ETS-1 pathway, miRs potentially targeting ETS-1 were predicted. As shown in **Figure 1D**, among these miRs, miR-338-5p had the highest expression levels in astrocytoma para-tumor tissues according to TCGA data. Moreover, the other miRs, miR-377-3p, miR-330-5p, miR-875-5p, miR-204-3p, miR-326, and miR-518c-5p, miR-495-3p, were almost indetectable in the astrocytoma clinical specimens (**Figure 1E**). The potential targeting site of miR-338-5p-5p in the 3’UTR of EST-1 is shown as a schematic diagram in **Figure 1F**. Based on these results, miR-338-5p-5p was chosen for the subsequent experiments.

The expression levels of miR-338-5p-5p in astrocytoma clinical specimens are shown in **Figure 1G**. The expression level of miR-338-5p-5p (detected as has-pre-miR-338-5p) in clinical specimens gradually decreased from grade I to grade IV, with the highest expression level in grade I (**Figure 1H**). Moreover, the expression level of miR-338-5p-5p was negatively correlated with that of ETS-1 (**Figure 1I**) and invasion-related genes downstream of ETS-1, MMP1 (**Figure 1J**) and MMP9 (**Figure 1K**). These results indicate that deficiency of miR-338-5p-5p would promote the aberrant activation of ETS-1 pathway.

### Hypermethylation of miR-338-5p-5p Mediated by DNMT-1 Is Associated With miR-338-5p-5p Deficiency and Aberrant Activation of the ETS-1 Pathway

**Figure 2:**
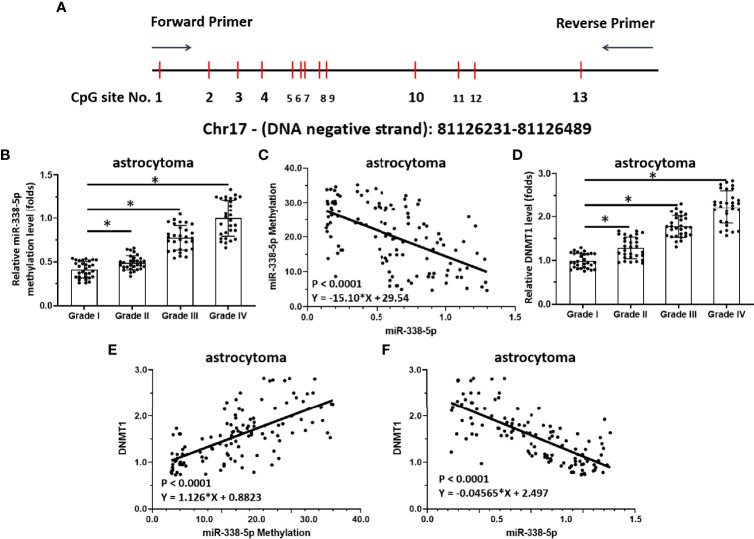
Methylation of miR-338-5p is correlated with disease severity in human astrocytoma patients. **(A)** Chosen sequences of the promoter region of miR-338-5p and the CpG islands/sites. There are 13 CpGs site located in this region (the 250bp amplicon closed to the transcription start site). **(B–D)** Scatter histogram showing methylation rates of miR-338-5p in human astrocytoma specimens **(B)**, correlation between miR-338-5p expression levels and the methylation rates of its promoter **(C)**, and expression levels of DNMT-1 in grade I–IV astrocytoma determined by qPCR **(D)**. **(E, F)** Scatter plots showing the correlation of methylation rates of the miR-338-5p promoter with DNMT-1 expression **(E)**, or miR-338-5p with DNMT-1. The statistical analysis was performed by student T-test without two-tails **(B, D)** and linear regression **(C, E, F)** by SPSS statistical software. *P < 0.05.

Next, the methylation rate of the miR-338-5p-5p promoter region was examined as a potential mechanism of miR-338-5p-5p deficiency. As shown in **Figure 2A**, there is a CpG site in the promoter region of the selected miR-338-5p-5p. The methylation rates of the miR-338-5p-5p promoter in clinical specimens gradually increased from grade I to grade IV, with the highest level in grade IV (**Figure 2B**). The expression of miR-338-5p-5p was negatively correlated with the methylation rate of its promoter region (**Figure 2C**). Given that the DNA methylation of tumor suppressors is mediated by DNMT1, the expression of DNMT-1 and its association with miR-338-5p-5p were examined. As shown in **Figure 2D**, the expression level of DNMT1 in clinical specimens gradually increased with severity, with the highest level of DNMT1 found in grade IV samples. Moreover, the DNMT-1 level was positively correlated with the methylation rate of the miR-338-5p promoter region (**Figure 2E**) and negatively correlated with expression levels of miR-338-5p in clinical specimens (**Figure 2F**). These results indicate that DNMT-1 contributes to the deficiency of miR-338-5p-5p via enhancing its promoter’s methylation.

### Knockdown of DNMT1 Enhanced the Expression of miR-338-5p-5p and Repressed the Expression of ETS1 in Astrocytoma Cells

**Figure 3:**
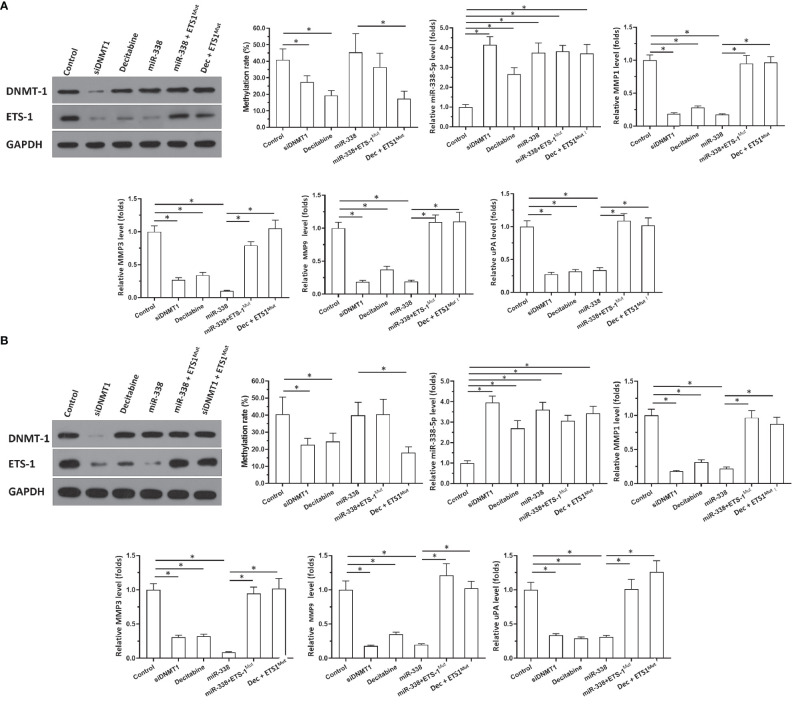
Effects of DNMT-1/miR-338-5p on the expression of ETS-1 in U87 or U251 cells. U87 **(A)** and U251 **(B)** cells were transfected with vectors. Then, cells were harvested for western blotting or qPCR. Western blot images show protein levels of DNMT-1 and ETS-1. Histograms show the methylation rates of the miR-338-5p promoter, miR-338-5p levels, and expression levels of MMP1, MMP3, MMP9, and uPA. The statistical analysis was performed by student T-test without two-tails. *P < 0.05.

To examine the effects of DNMT1 knockdown on the miR-338-5p-5p/ETS-1 axis and confirm the specificity of miR-338-5p-5p’s targeting of ETS-1, qPCR and western blot assays were performed. As shown in **Figures 3A, B**, knockdown of DNMT1 inhibited the expression of DNMT1 and ETS-1 in U87 and U251 cells. Treatment with decitabine, an antagonist of DNMT1, inhibited the expression of ETS-1 but not that of DNMT1 (**Figures 3A, B**). Moreover, treatment with decitabine or a small interfering RNA (siRNA) targeting DNMT1 (siDNMT1) decreased the methylation rate of the miR-338-5p-5p promoter and enhanced the expression of miR-338-5p-5p in U251 cells (**Figures 3A, B**).

The specificity of miR-338-5p-5p toward ETS-1 was also confirmed. As shown in **Figure 3**, overexpression of miR-338-5p-5p did not affect the expression of DNMT1 or the methylation rate of the miR-338-5p-5p promoter, but it decreased the expression of ETS-1 in U87 (**Figure 3A**) and U251 cells (**Figure 3B**). Transfection of ETS-1 with a mutated miR-338-5p-5p targeting site (ETS-1^Mut^) almost completely blocked the effects of miR-338-5p-5p or decitabine treatment (**Figures 3A, B**). **Figures 3A, B** show the western blotting results, and histograms (mean ± SD) of the methylation rates of the miR-338-5p-5p promoter and the expression levels of miR-338-5p-5p and invasion-related genes downstream of ETS-1 (MMP1, MMP3, MMP9, and uPA). These results indicate that knockdown of DNMT1 or inhibition of its activity repressed the activation of the ETS-1 pathway *via* enhancing the expression of miR-338-5p-5p, which targets the 3’UTR of ETS1 in astrocytoma cells.

### Inhibition of DNMT1 Activity Inhibited Invasion of Astrocytoma Cells *In Vitro*

In addition to the above experiments, which demonstrated the effects of DNMT1 on the miR-338-5p-5p/ETS-1 axis, the transwell assays were performed to examine the effects of DNMT1 on the invasion of astrocytoma cells. As shown as **Supplemental Figure 1A**, treatment with decitabine inhibited the invasion of U87 cells *in vitro*, whereas transfection of ETS-1^Mut^ almost completely blocked the effects of decitabine on U87 cells. Similar results were obtained in U251 cells (**Supplemental Figure 1B**). Therefore, inhibition of DNMT1 activity inhibited the invasion of astrocytoma cells *in vitro*.

### Inhibition of DNMT1 Activity Inhibited Subcutaneous Growth of Astrocytoma Cells

**Figure 4:**
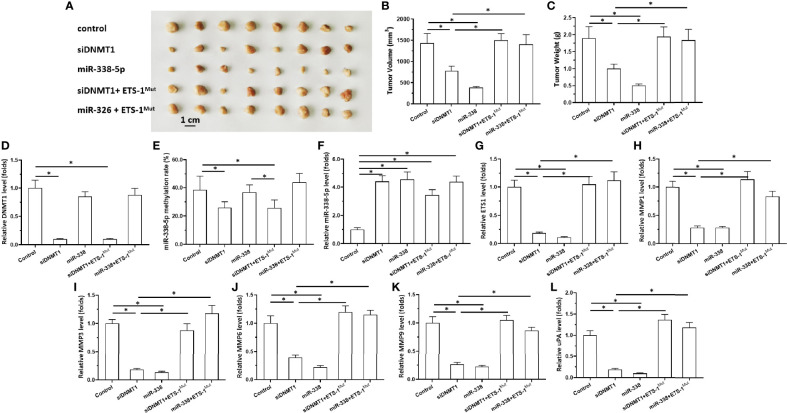
Effects of DNMT-1/miR-338-5p on the subcutaneous growth of U87 cells. U87 cells were transfected with vectors, then injected subcutaneously into nude mice. **(A)** Images of the subcutaneous tumor tissues. **(B)** Tumor volumes; **(C)** tumor weights. **(D–L)** Histograms showing expression levels of DNMT-1 **(D)**, methylation rates of the miR-338-5p promoter **(E)**, and levels of miR-338-5p **(F)**, ETS-1 **(G)**, MMP1 **(H)**, MMP3 **(I)**, MMP6 **(J)**, MMP9 **(K)**, and uPA **(L)** in subcutaneous tumor tissues. The statistical analysis was performed by student T-test without two-tails. *P < 0.05.

**Figure 5:**
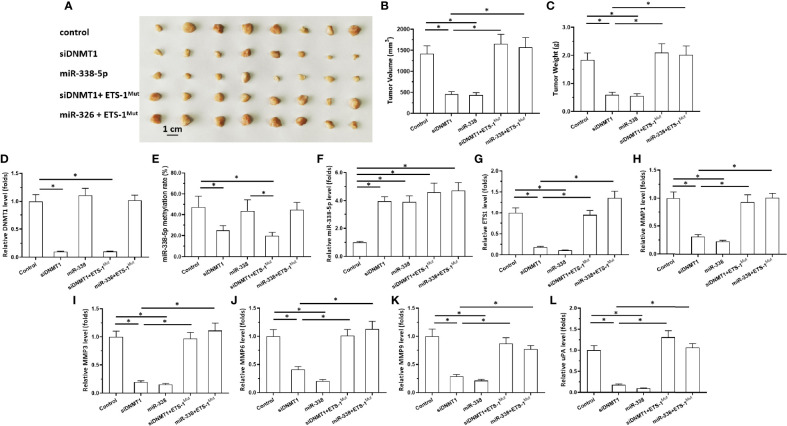
Effects of DNMT-1/miR-338-5p on the subcutaneous growth of U251 cells. U251 cells were transfected with vectors, then injected subcutaneously into nude mice. **(A)** Images of the subcutaneous tumor tissues. **(B)** Tumor volumes; **(C)** tumor weights. **(D–L)** Histograms showing expression levels of DNMT-1 **(D)**, methylation rates of the miR-338-5p promoter **(E)**, and levels of miR-338-5p **(F)**, ETS-1 **(G)**, MMP1 **(H)**, MMP3 **(I)**, MMP6 **(J)**, MMP9 **(K)**, and uPA **(L)** in subcutaneous tumor tissues. The statistical analysis was performed by student T-test without two-tails. *P < 0.05.

Next, the effects of DNMT1 knockdown on *in vivo* growth of astrocytoma cells was examined using a nude mice model. The cells were transfected with vectors and then harvested for the subcutaneous tumor model (**Figures 4** and **5**). As shown in **Figure 4**, U87 cells formed subcutaneous tissues in nude mice. Transfection of siDNMT1 or miR-338-5p inhibited the subcutaneous growth of U87 cells (**Figures 4A–C**). Transfection of ETS-1^Mut^ almost completely blocked the inhibitory effect of siDNMT1 or miR-338-5p on the subcutaneous growth of U251 cells (**Figures 4A–C**). The specificity of the effects of the DNMT1/miR-338-5p-5p/ETS1 axis on the subcutaneous growth of U87 cells was confirmed by the expression levels of factors related to the DNMT1/miR-338-5p-5p/ETS1 axis and the methylation rates of the miR-338-5p-5p promoter (**Figures 4D–L**). Similar results were obtained in U251 cells (**Figure 5**). Therefore, inhibition of DNMT1 activity inhibited the subcutaneous growth of astrocytoma cells.

### The Effect of miR-338-5p’s Promoter Methylation by Other Inhibitors to Discard Other Mechanisms

The above experiments mainly focused on the usage of Decitabine, the other inhibitors, Azacitidine or TSA, were also used to discard other mechanisms. As shown in **Supplemental Figure 2**, Decitabine or Azacitidine inhibited the methylation of miR-338-5p promoter in U251 or U87 cells in a dose dependent manner, and Decitabine is better than Azacitidine on the methylation of miR-338-5p promoter (**Supplemental Figure 2**). Treatment of TSA did not affect the methylation of miR-338-5p promoter (**Supplemental Figure 2**). These results further confirmed the specificity of DNMT-1 on miR-338-5p.

## Discussion

Accumulating data indicate that prognosis and therapeutic indications vary significantly among different grades of astrocytoma (1–5). In the present work, aberrant activation of the ETS-1 pathway was identified in high-grade astrocytomas compared with low-grade astrocytomas. Ren et al. in 2016 showed that some extracellular matrix (ECM)-related proteins, including MMPs, were upregulated in high-grade astrocytomas (13). Our results were consistent with those of Ren et al. and would reveal the potential mechanism underlying the high expression of MMPs, that is, the high expression of ETS-1 in astrocytoma tissue may eventually participate in the high expression of MMPs. The highly aggressive characteristics of malignant tumor cells are closely related to disease progression; the main mechanism of this process is that malignant tumor cells destroy ECM through MMPs and so on to reshape the basic structure of tissues, thereby promoting both the growth of tumor cells and their transfer to other locations (44–48). As a transcription factor, ETS-1 can mediate the transcription of MMPs, etc. The results of this study indicate that the high expression of ETS-1 in astrocytoma and other tissues represents a possible mechanism of disease progression (49–51). Owing to the intercranial position of astrocytoma tumors, it was difficult to obtain many adjacent non-tumor tissues. Instead, we used data of glioma non-tumor tissues from the TCGA database. Nevertheless, our results confirmed the expression levels of ETS-1 in tissue specimens of different astrocytoma grades, reflecting the role of ETS-1 in astrocytoma. ETS-1 is an ideal target for astrocytoma therapy. Jie et al. in 2021 reported a small-molecule inhibitor of ETS-1, which could be used in anti-tumor therapy targeting ETS-1 (17). Our results firstly revealed the relationship between ETS-1 expression and disease progression in clinical specimens. After that, the possible mechanism of the high expression of ETS-1 in astrocytoma was initially explored, and a series of research techniques (Transwell, nude mouse subcutaneous tumor formation experiment) were used to detect the invasion of U87 and U251 cells *in vitro* by ETS-1 and its upstream pathways and the influence of these factors was also revealed in tumor formation of nude mice.

**Figure 6:**
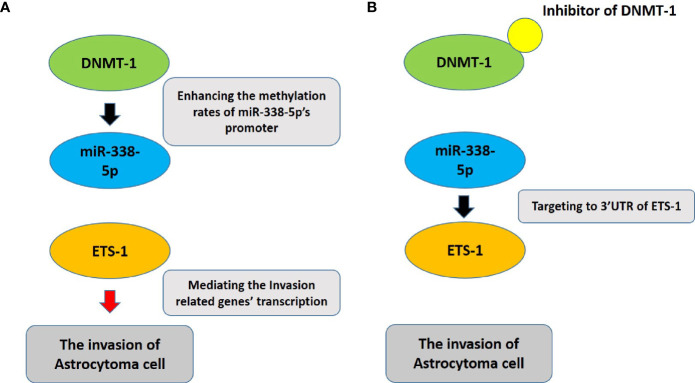
The proposal model of the presence work. **(A)** DNMT-1 inhibits the expression of miR-338-5p by up-regulating the promoter methylation rate of miR-338-5p, thereby up-regulating the expression level of ETS-1, and ultimately promotes the invasion of astrocytoma cells. **(B)** The usage of small molecule inhibitors of DNMT-1 can inhibit the activity of DNMT-1. At this time, the methylation level of the promoter region of miR-338-5p is down-regulated, and correspondingly the expression level of miR-338-5p is up-regulated, eventually leading to the down-regulation of ETS-1 Pathway’s activity to inhibit the invasion of astrocytoma cells.

Furthermore, this study investigated the mechanism underlying the high expression of ETS-1 in astrocytoma tissue. Loss of miR-338-5p-5p expression is a possible cause of ETS-1 overexpression (**Figure 6**). Methylation of the promoter region of miR-338-5p-5p can also lead to low levels of miR-338-5p-5p in astrocytoma. The miRNA is an important type of small molecule non-coding RNA, which can not only be used as an anti-tumor treatment strategy (for example, in this study, the entire sequence of hsa-pre-miR-338-5p was prepared as rotavirus/lentivirus), but also the deficiency of miRs would be the possible mechanisms for the aberrant expression of its target gene (some pro-oncogene) in malignant tumor tissues (26, 52–54). In terms of molecular mechanism, miRNA is used in the 3’UTR region of its target gene through a sequence-specific mechanism (55–59). For this reason, it is necessary to first predict based on a sequence-specific method, and then conduct confirmation of miRs on a specific target gene. In this study, miRDB was first used for prediction, and it was found that there is a miR-338-5p site in the 3’UTR region of ETS-1. The precursor molecule of miR-338 (has-pre-miR-338) was further prepared into lentiviral particles and transfected into U251 and U87 cells, and it was found that miR-338-5p can inhibit the expression of ETS-1. At the same time construct the ETS-1 full-length expression vector containing 3’UTR and the ETS-1 expression vector containing the mutant miR-338-5p site (at this time, the ATATTGT sequence in ETS-1 3’UTR is mutated to CGCGGTG). Overexpression of miR-338-5p can inhibit the expression of wild-type ETS-1 in U251 and U87 cells, but cannot affect the expression of ETS-1 mutants. These all confirmed the effect of miR-338-5p on ETS-1

Since the methylation of the promoter region is an important mechanism for the loss of specific gene expression, the methylation of the miR-338-5p promoter region was tested for this reason (10, 60, 61). The results of this study found that the methylation level of the promoter region of miR-338-5p increased as the stage of astrocytoma increased. At the same time, the methylation rate of miR-338-5p promoter region is also negatively correlated with the expression of ETS-1. To analyze the possible mechanism of miR-338-5p promoter region, the high expression of DNMT-1 in astrocytoma is the possible mechanism of miR-338-5p promoter region hypermethylation. DNMT1 mediates the methylation of the promoter region of miR-338-5p-5p, and the effect of DNMT1 on miR-338-5p-5p has been confirmed in clinical specimens. The use of a siRNA or antagonist against DNMT1 in U251 and U87 cells was shown to decrease the methylation level of the promoter region of miR-338-5p-5p, upregulate miR-338-5p expression, and finally downregulate ETS-1 expression. Among the four types of DNA methyltransferases, DNMT1 not only participates in the normal methylation process but also induces the silencing of tumor suppressors *via* hypermethylation of their promoter regions (62, 63). The main function of DNMT3 (DNMT3a and DNMT3b) is *de novo* methylation in the early stage of embryonic development, whereas DNMT2 (also known as tRNA aspartic acid methyltransferase 1) is classified as an RNA methylase (64–66). Therefore, our results mainly focus on DNMT1.

In the present work, the methylation rate of the miR-338-5p-5p promoter was examined using a BSP-NGS method, in contrast to the MSP method traditionally used in methylation research (28). MSP uses sulfite treatment followed by PCR; the PCR product is cloned into a T-vector to transform Escherichia coli, which are plated to form single-cell colonies, and single colonies are picked for first-generation sequencing analysis. The main limitation of the MSP strategy is that it uses only one pair of primers to amplify one amplicon, which is then transformed to form a single bacterial colony, representing one cell. Thus, it can only be used to qualitatively determine whether there is a methylation modification at a given CpG site. The BSP-NGS methods used in the present work can overcome the shortcomings of the MSP method: after sulfite treatment and high-fidelity PCR, the PCR product (amplicon) is directly subjected to high-throughput sequencing, and the DNA CpG positions of all cells in the astrocytoma tissue are detected. The sequencing results directly reflect the methylation rate of the selected CpG site. The Ion 318™ Chip v2 BC chip used in this study can sequence 96 samples at a time. It can not only provide new research methods for methylation-related research, but also provides new ideas for the epigenetic-related research (67–70).

Based on the factors that ETS-1 is not only an important regulator of human cancerous cells’ proliferation, but also enhancing the invasive ability of cells, the transwell assays were performed (16, 18, 71). The results were shown as the images of the invaded cells or the quantitative results of the images. The basic principle of Transwell is to use 24-well plates as the outer chambers and the inner chambers (72). The bottom of the inner chamber is a thin film with evenly distributed 8μm diameter holes (45). First, 10% concentration of ECM gel (the ECM gel produced by Sigma, diluted with serum-free DMEM in a ratio of 1:10) is added to the bottom of the inner chambers and a thin film is formed to simulate the basement membrane of the tissue (45). After that, the cells are prepared as a single cell suspension (the cell suspension is prepared in DMEM without serum at this time) and then added to the inner chamber (45). The outer chambers (that is, the wells of the 24-well plates) are added with DMEM containing 20% FBS, and then the inner chambers are added to the 24-well cell plates (45). At this time, the cells in the inner chambers will be driven and attracted by the FBS in the outer chambers (this is a standard chemotaxis), migrate from the inner chamber to the outer chamber, and finally distribute on the outside of the bottom of the inner chambers. Since there is a layer of ECM gel inside the bottom of the inner chambers to mimic the tissue basement membrane, the cells need to destroy the ECM gel before they can migrate from the inside of the inner chambers to the outside (45). This process perfectly mimics the invasion of tumor cells. In this study, through the Transwell experiment, the use of siRNA of DNMT-1 or hsa-pre-miR-338 can significantly inhibit the *in vitro* invasion of U251 or U87 cells; transfection of ETS-1 mutants can inhibit the inhibitory roles of miR-338 or siDNMT on U251 or U87 cells’ *in vitro* invasion.

Decitabine is a common and typical DNMT-1 inhibitor, which has a good inhibitory activity on the activity of DNMT-1 (73–75). While using Decitabine, this study also used inhibitors of other mechanisms. Azacitidine (5-Azacytidine, 5-AzaC, Ladakamycin, AZA, 5-Aza, CC-486) is a nucleoside analog of cytidine, which inhibits DNA methylation by binding to DNA methyltransferase in competition with cytidine (75–78). Our results show that Azacitidine can also down-regulate the promoter methylation rate of miR-338-5p, but the activity of Decitabine is better than that of Azacitidine (that is, the inhibitory activity of Decitabine is higher than that of Azacitidine at the same concentration). This may due to the fact that Decitabine directly inhibits DNMT-1, while Azacitidine needs to compete with cytidine to bind to DNMT-1. Trichostatin A (TSA) is an HDAC inhibitor (79–81), which has nothing to do with DNA methylation. TSA treatment of U251 or U87 cells does not affect the methylation rate of the miR-338-5p promoter region in the cells. This result further confirms the specificity of DNMT-1/miR-338-5p/ETS-1 axis.

## Data Availability Statement

The original contributions presented in the study are included in the article/**Supplementary Material**. Further inquiries can be directed to the corresponding authors.

## Ethics Statement

The studies involving human participants were reviewed and approved by Ethics Committee of the Second Affiliated Hospital of Dalian Medical University. The patients/participants provided their written informed consent to participate in this study. The animal study was reviewed and approved by Animal Ethics Committee of the Second Affiliated Hospital of Dalian Medical University.

## Author Contributions

JW, LM, and DW: concept, design, statistics, data collection, manuscript writing, final approval. CH: design, statistics, data collection. JY: concept, data collection. JW and JY: statistics, manuscript writing. LT: statistics, data collection. LM and LT: statistics, data collection. DW: concept, design, statistics, data collection, manuscript writing, final approval. All authors contributed to the article and approved the submitted version.

## Conflict of Interest

The authors declare that the research was conducted in the absence of any commercial or financial relationships that could be construed as a potential conflict of interest.

## Publisher’s Note

All claims expressed in this article are solely those of the authors and do not necessarily represent those of their affiliated organizations, or those of the publisher, the editors and the reviewers. Any product that may be evaluated in this article, or claim that may be made by its manufacturer, is not guaranteed or endorsed by the publisher.

## Supplementary Material

Supplementary Figure 1 Effects of DNMT-1/miR-338-5p on invasion of U87 and U251 cells *in vitro*. U87 **(A)** and U251 **(B)** cells were transfected with vectors. Then, cells were harvested for *in vitro* transwell experiments. Images of the invaded cells are shown, as well as quantitative results. The statistical analysis was performed by student T-test without two-tails. *P < 0.05.

Supplementary Figure 2 The effect on inhibitors on the methylation rates of miR-338-5p promoter in U87 and U251 cells. The U87 **(A-C)** and U251 cells **(D-F)** cells were treated with the indicated concentrations of Decitabine **(A, D)**, Azacitidine **(B, E)** or TSA **(C, F)**, was harvested for the BSP-NGS. The results were shown as histogram of miR-338-5p promoter’s methylation rates (mean ± SD). The statistical analysis was performed by student T-test without two-tails. P < 0.05.

## References

[1] Stupp R Lukas RV Hegi ME. Improving Survival in Molecularly Selected Glioblastoma. Lancet (2019) 393(10172):615–7. doi: 10.1016/S0140-6736(18)33211-2 30782332

[2] Franz DN Belousova E Sparagana S Bebin EM Frost M Kuperman R. Efficacy and Safety of Everolimus for Subependymal Giant Cell Astrocytomas Associated With Tuberous Sclerosis Complex (EXIST-1): A Multicentre, Randomised, Placebo-Controlled Phase 3 Trial. Lancet (2013) 381(9861):125–32. doi: 10.1016/S0140-6736(12)61134-9 23158522

[3] Draaisma K Chatzipli A Taphoorn M Kerkhof M Weyerbrock A Sanson M. Molecular Evolution of IDH Wild-Type Glioblastomas Treated With Standard of Care Affects Survival and Design of Precision Medicine Trials: A Report From the EORTC 1542 Study. J Clin Oncol (2020) 38(1):81–99. doi: 10.1200/JCO.19.00367 31743054

[4] Huang B Li X Li Y Zhang J Zong Z Zhang H. Current Immunotherapies for Glioblastoma Multiforme. Front Immunol (2021) 11:603911. doi: 10.3389/fimmu.2020.603911 33767690 PMC7986847

[5] Barbagallo GMV Altieri R Garozzo M Maione M Di Gregorio S Visocchi M. High Grade Glioma Treatment in Elderly People: Is It Different Than in Younger Patients? Analysis of Surgical Management Guided by an Intraoperative Multimodal Approach and Its Impact on Clinical Outcome. Front Oncol (2021) 10:631255. doi: 10.3389/fonc.2020.631255 33718122 PMC7943843

[6] Altieri R Certo F Rocca G Melcarne A Garbossa D Bianchi A. Radiological Evaluation of Ex Novo High Grade Glioma: Velocity of Diametric Expansion and Acceleration Time Study. Radiol Oncol (2020) 55(1):26–34. doi: 10.2478/raon-2020-0071 33885243 PMC7877266

[7] Forjaz G Barnholtz-Sloan JS Kruchko C Siegel R Negoita S Ostrom QT. An Updated Histology Recode for the Analysis of Primary Malignant and Nonmalignant Brain and Other Central Nervous System Tumors in the Surveillance, Epidemiology, and End Results Program. Neurooncol Adv (2020) 3(1):vdaa175. doi: 10.1093/noajnl/vdaa175 33506208 PMC7813198

[8] Djurovic Z Jovanovic V Obrenovic R Djurovic B Soldatovic I Vranic A. The Importance of the Blood Levels of Homocysteine, Folate and Vitamin B12 in Patients With Primary Malignant Brain Tumors. J BUON (2020) 25(6):2600–7. 33455102

[9] Yang J Yang Q. Identification of Core Genes and Screening of Potential Targets in Glioblastoma Multiforme by Integrated Bioinformatic Analysis. Front Oncol (2021) 10:615976. doi: 10.3389/fonc.2020.615976 33718116 PMC7943725

[10] Li DM Chen QD Wei GN Wei J Yin JX He JH. Hypoxia-Induced miR-137 Inhibition Increased Glioblastoma Multiforme Growth and Chemoresistance Through Lrp6. Front Oncol (2021) 10:611699. doi: 10.3389/fonc.2020.611699 33718112 PMC7946983

[11] Zheng L Zhou ZR Yu Q Shi M Yang Y Zhou X. The Definition and Delineation of the Target Area of Radiotherapy Based on the Recurrence Pattern of Glioblastoma After Temozolomide Chemoradiotherapy. Front Oncol (2021) 10:615368. doi: 10.3389/fonc.2020.615368 33692942 PMC7937883

[12] Miyake K Suzuki K Ogawa T Ogawa D Hatakeyama T Shinomiya A. Multiple Positron Emission Tomography Tracers for Use in the Classification of Gliomas According to the 2016 World Health Organization Criteria. Neurooncol Adv (2020) 3(1):vdaa172. doi: 10.1093/noajnl/vdaa172 33681765 PMC7920529

[13] Ren T Lin S Wang Z Shang A. Differential Proteomics Analysis of Low- and High-Grade of Astrocytoma Using iTRAQ Quantification. Onco Targets Ther (2016) 9:5883–5895. doi: 10.2147/OTT.S111103 27713642 PMC5045242

[14] Birzu C Tran S Bielle F Touat M Mokhtari K Younan N. Leptomeningeal Spread in Glioblastoma: Diagnostic and Therapeutic Challenges. Oncologist (2020) 25(11):e1763–76. doi: 10.1634/theoncologist.2020-0258 PMC7648332 33394574

[15] Wang Y Cai R Wang P Huang C Zhang C Liu Z. FAM46A Expression is Elevated in Glioblastoma and Predicts Poor Prognosis of Patients. Clin Neurol Neurosurg (2021) 201:106421. doi: 10.1016/j.clineuro.2020.106421 33370626

[16] Zhou Q Liu M Shao T Xie P Zhu S Wang W. TPX2 Enhanced the Activation of the HGF/ETS-1 Pathway and Increased the Invasion of Endocrine-Independent Prostate Carcinoma Cells. Front Oncol (2021) 11:618540. doi: 10.3389/fonc.2021.618540 34123781 PMC8193931

[17] Jie Y Liu G Mingyan E Li Y Xu G Guo J. Novel Small Molecule Inhibitors of the Transcription Factor ETS-1 and Their Antitumor Activity Against Hepatocellular Carcinoma. Eur J Pharmacol (2021) 906:174214. doi: 10.1016/j.ejphar.2021.174214 34116044

[18] Du Y Shi X Ma W Wen P Yu P Wang X. Phthalates Promote the Invasion of Hepatocellular Carcinoma Cells by Enhancing the Interaction Between Pregnane X Receptor and E26 Transformation Specific Sequence 1. Pharmacol Res (2021) 169:105648. doi: 10.1016/j.phrs.2021.105648 33965509

[19] Min J Pan X Lv G. The circRNA Circ_0000291 Acts as a Sponge of microRNA 326 to Regulate E26 Transformation-Specific Sequence-1 Expression and Promote Breast Cancer Progression. Pathol Int (2020) 70(12):953–64. doi: 10.1111/pin.13011 32869935

[20] Mao P Brown AJ Esaki S Lockwood S Poon GMK Smerdon MJ. ETS Transcription Factors Induce a Unique UV Damage Signature That Drives Recurrent Mutagenesis in Melanoma. Nat Commun (2018) 9(1):2626. doi: 10.1038/s41467-018-05064-0 29980679 PMC6035183

[21] Li W Yan P Meng X Zhang J Yang Y. The microRNA Cluster miR-214/miR-3120 Prevents Tumor Cell Switching From an Epithelial to a Mesenchymal-Like Phenotype and Inhibits Autophagy in Gallbladder Cancer. Cell Signal (2021) 80:109887. doi: 10.1016/j.cellsig.2020.109887 33340658

[22] Visci G Tolomeo D Agostini A Traversa D Macchia G Storlazzi CT. CircRNAs and Fusion-circRNAs in Cancer: New Players in an Old Game. Cell Signal (2020) 75:109747. doi: 10.1016/j.cellsig.2020.109747 32860952

[23] Li B Feng F Jia H Jiang Q Cao S Wei L. Rhamnetin Decelerates the Elimination and Enhances the Antitumor Effect of the Molecular-Targeting Agent Sorafenib in Hepatocellular Carcinoma Cells *via* the miR-148a/PXR Axis. Food Funct (2021) 12(6):2404–17. doi: 10.1039/d0fo02270e 33570057

[24] Deng N Guo R Zheng B Li T Liu RH. IRS-1/PI3K/Akt Pathway and miRNAs are Involved in Whole Grain Highland Barley (Hordeum Vulgare L.) Ameliorating Hyperglycemia of Db/Db Mice. Food Funct (2020) 11(11):9535–46. doi: 10.1039/d0fo01990a 33104141

[25] Liu J Li Y Xue L Fan M Nie C Wang Y. Circulating miR-27a-3p as a Candidate for a Biomarker of Whole Grain Diets for Lipid Metabolism. Food Funct (2020) 11(10):8852–65. doi: 10.1039/d0fo00830c 32975270

[26] Chong ZX Yeap SK Ho WY. Unraveling the Roles of miRNAs in Regulating Epithelial-To-Mesenchymal Transition (EMT) in Osteosarcoma. Pharmacol Res (2021) 172:105818. doi: 10.1016/j.phrs.2021.105818 34400316

[27] Shopit A Li X Tang Z Awsh M Shobet L Niu M. miR-421 Up-Regulation by the Oleanolic Acid Derivative K73-03 Regulates Epigenetically SPINK1 Transcription in Pancreatic Cancer Cells Leading to Metabolic Changes and Enhanced Apoptosis. Pharmacol Res (2020) 161:105130. doi: 10.1016/j.phrs.2020.105130 32818653

[28] Ma Y Chai N Jiang Q Chang Z Chai Y Li X. DNA Methyltransferase Mediates the Hypermethylation of the microRNA 34a Promoter and Enhances the Resistance of Patient-Derived Pancreatic Cancer Cells to Molecular Targeting Agents. Pharmacol Res (2020) 160:105071. doi: 10.1016/j.phrs.2020.105071 32659427

[29] Virakul S Somparn P Pisitkun T van der Spek PJ Dalm VASH Paridaens D. Integrative Analysis of Proteomics and DNA Methylation in Orbital Fibroblasts From Graves’ Ophthalmopathy. Front Endocrinol (Lausanne) (2021) 11:619989. doi: 10.3389/fendo.2020.619989 33658982 PMC7919747

[30] He W Gong S Wang X Dong X Cheng H. DNA Methylation Integratedly Modulates the Expression of Pit-Oct-Unt Transcription Factors in Esophageal Squamous Cell Carcinoma. J Cancer (2021) 12(6):1634–43. doi: 10.7150/jca.49231 PMC7890322 33613750

[31] Wang Y Liu S Chen Q Ren Y Li Z Cao S. Novel Small Molecular Inhibitor of Pit-Oct-Unc Transcription Factor 1 Suppresses Hepatocellular Carcinoma Cell Proliferation. Life Sci (2021) 277:119521. doi: 10.1016/j.lfs.2021.119521 33891940

[32] Zou XZ Zhou XH Feng YQ Hao JF Liang B Jia MW. Novel Inhibitor of OCT1 Enhances the Sensitivity of Human Esophageal Squamous Cell Carcinoma Cells to Antitumor Agents. Eur J Pharmacol (2021) 907:174222. doi: 10.1016/j.ejphar.2021.174222 34087221

[33] Hiramatsu-Asano S Sunahori-Watanabe K Zeggar S Katsuyama E Mukai T Morita Y. Deletion of Mir223 Exacerbates Lupus Nephritis by Targeting S1pr1 in Faslpr/lpr Mice. Front Immunol (2021) 11:616141. doi: 10.3389/fimmu.2020.616141 33574820 PMC7871001

[34] Ge X Jiang W Jiang Y Lv X Liu X Wang X. Expression and Importance of TMED2 in Multiple Myeloma Cells. Cancer Manag Res (2020) 12:12895–903. doi: 10.2147/CMAR.S278570 PMC7751311 33364837

[35] Xue Y Li PD Tang XM Yan ZH Xia SS Tian HP. Cytochrome C Oxidase Assembly Factor 1 Homolog Predicts Poor Prognosis and Promotes Cell Proliferation in Colorectal Cancer by Regulating PI3K/AKT Signaling. Onco Targets Ther (2020) 13:11505–16. doi: 10.2147/OTT.S279024 PMC7667209 33204105

[36] Cao Y Trillo-Tinoco J Sierra RA Anadon C Dai W Mohamed E. ER Stress-Induced Mediator C/EBP Homologous Protein Thwarts Effector T Cell Activity in Tumors Through T-Bet Repression. Nat Commun (2019) 10(1):1280. doi: 10.1038/s41467-019-09263-1 30894532 PMC6426975

[37] Fan Z Yang J Zhang D Zhang X Ma X Kang L. The Risk Variant Rs884225 Within EGFR Impairs miR-103a-3p’s Anti-Tumourigenic Function in non-Small Cell Lung Cancer. Oncogene (2019) 38(13):2291–304. doi: 10.1038/s41388-018-0576-6 30470824

[38] Chi X Luo W Song J Li B Su T Yu M. Kindlin-2 in Sertoli Cells is Essential for Testis Development and Male Fertility in Mice. Cell Death Dis (2021) 12(6):604. doi: 10.1038/s41419-021-03885-4 34117213 PMC8196014

[39] Yang S Xu W Liu C Jin J Li X Jiang Y. LATS1 K751 Acetylation Blocks Activation of Hippo Signalling and Switches LATS1 From a Tumor Suppressor to an Oncoprotein. Sci China Life Sci (2021). doi: 10.1007/s11427-020-1914-3 33945069

[40] Meng C Zhan J Chen D Shao G Zhang H Gu W. The Deubiquitinase USP11 Regulates Cell Proliferation and Ferroptotic Cell Death *via* Stabilization of NRF2 USP11 Deubiquitinates and Stabilizes Nrf2. Oncogene (2021) 40(9):1706–20. doi: 10.1038/s41388-021-01660-5 33531626

[41] Zhang Z Hou Y Wang Y Gao T Ma Z Yang Y. Regulation of Adipocyte Differentiation by METTL4, a 6 mA Methylase. Sci Rep (2020) 10(1):8285. doi: 10.1038/s41598-020-64873-w 32427889 PMC7237444

[42] Chu W Zhang X Qi L Fu Y Wang P Zhao W. The EZH2-PHACTR2-AS1-Ribosome Axis Induces Genomic Instability and Promotes Growth and Metastasis in Breast Cancer. Cancer Res (2020) 80(13):2737–50. doi: 10.1158/0008-5472.CAN-19-3326 32312833

[43] Song J Wang T Chi X Wei X Xu S Yu M. Kindlin-2 Inhibits the Hippo Signaling Pathway by Promoting Degradation of MOB1. Cell Rep (2019) 29(11):3664–3677.e5. doi: 10.1016/j.celrep.2019.11.035 31825843

[44] Guo Z Dai Y Hu W Zhang Y Cao Z Pei W. The Long Noncoding RNA CRYBG3 Induces Aneuploidy by Interfering With Spindle Assembly Checkpoint *via* Direct Binding With Bub3. Oncogene (2021) 40(10):1821–35. doi: 10.1038/s41388-020-01601-8 PMC7946627 33564066

[45] Zhang P Ma X Song E Chen W Pang H Ni D. Tubulin Cofactor A Functions as a Novel Positive Regulator of ccRCC Progression, Invasion and Metastasis. Int J Cancer (2013) 133(12):2801–11. doi: 10.1002/ijc.28306 23740643

[46] Rajesh Y Banerjee A Pal I Biswas A Das S Dey KK. Delineation of Crosstalk Between HSP27 and MMP-2/MMP-9: A Synergistic Therapeutic Avenue for Glioblastoma Management. Biochim Biophys Acta Gen Subj (2019) 1863(7):1196–209. doi: 10.1016/j.bbagen.2019.04.015 31028823

[47] Peng L Wen L Shi QF Gao F Huang B Meng J. Scutellarin Ameliorates Pulmonary Fibrosis Through Inhibiting NF-κb/NLRP3-Mediated Epithelial-Mesenchymal Transition and Inflammation. Cell Death Dis (2020) 11(11):978. doi: 10.1038/s41419-020-03178-2 33188176 PMC7666141

[48] Qi J Zhou N Li L Mo S Zhou Y Deng Y. Ciclopirox Activates PERK-Dependent Endoplasmic Reticulum Stress to Drive Cell Death in Colorectal Cancer. Cell Death Dis (2020) 11(7):582. doi: 10.1038/s41419-020-02779-1 32719342 PMC7385140

[49] Srivastava N Bishnoi A Mehta S Rani S Kumar R Bhardwaj S. Aberrant ETS-1 Signalling Impedes the Expression of Cell Adhesion Molecules and Matrix Metalloproteinases in non-Segmental Vitiligo. Exp Dermatol (2020) 29(6):539–47. doi: 10.1111/exd.14107 32350934

[50] Nazir SU Kumar R Singh A Khan A Tanwar P Tripathi R. Breast Cancer Invasion and Progression by MMP-9 Through Ets-1 Transcription Factor. Gene (2019) 711:143952. doi: 10.1016/j.gene.2019.143952 31265880

[51] Yalim-Camci I Balcik-Ercin P Cetin M Odabas G Tokay N Sayan AE. ETS1 is Coexpressed With ZEB2 and Mediates ZEB2-Induced Epithelial-Mesenchymal Transition in Human Tumors. Mol Carcinog (2019) 58(6):1068–81. doi: 10.1002/mc.22994 30790340

[52] Chen Z Chu S Liang Y Xu T Sun Y Li M. miR-497 Regulates Fatty Acid Synthesis *via* LATS2 in Bovine Mammary Epithelial Cells. Food Funct (2020) 11(10):8625–36. doi: 10.1039/d0fo00952k 32935676

[53] He H Feng M Xu H Li X He Y Qin H. Total Triterpenoids From the Fruits of Chaenomeles Speciosa Exerted Gastroprotective Activities on Indomethacin-Induced Gastric Damage *via* Modulating microRNA-423-5p-Mediated TFF/NAG-1 and Apoptotic Pathways. Food Funct (2020) 11(1):662–79. doi: 10.1039/c9fo02322d 31895380

[54] Mirzaei S Mohammadi AT Gholami MH Hashemi F Zarrabi A Zabolian A. Nrf2 Signaling Pathway in Cisplatin Chemotherapy: Potential Involvement in Organ Protection and Chemoresistance. Pharmacol Res (2021) 167:105575. doi: 10.1016/j.phrs.2021.105575 33771701

[55] Wang G Lin F Wan Q Wu J Luo M. Mechanisms of Action of Metformin and its Regulatory Effect on microRNAs Related to Angiogenesis. Pharmacol Res (2021) 164:105390. doi: 10.1016/j.phrs.2020.105390 33352227

[56] Gu XQ Tang D Wan P Qin T Yang TH Wu J. Multiple microRNAs Regulate Tacrolimus Metabolism Through Cyp3a5. Pharmacol Res (2021) 164:105382. doi: 10.1016/j.phrs.2020.105382 33348024

[57] Kang W Wang Q Dai Y Wang H Wang M Wang J. Hypomethylation of PlncRNA-1 Promoter Enhances Bladder Cancer Progression Through the miR-136-5p/Smad3 Axis. Cell Death Dis (2020) 11(12):1038. doi: 10.1038/s41419-020-03240-z 33288752 PMC7721747

[58] Yang H Ren L Wang Y Bi X Li X Wen M. FBI-1 Enhanced the Resistance of Triple-Negative Breast Cancer Cells to Chemotherapeutic Agents *via* the miR-30c/PXR Axis. Cell Death Dis (2020) 11(10):851. doi: 10.1038/s41419-020-03053-0 33051436 PMC7554048

[59] Yang B Wang C Xie H Wang Y Huang J Rong Y. MicroRNA-3163 Targets ADAM-17 and Enhances the Sensitivity of Hepatocellular Carcinoma Cells to Molecular Targeted Agents. Cell Death Dis (2019) 10(10):784. doi: 10.1038/s41419-019-2023-1 31611551 PMC6791891

[60] Liu T Cai J Cai J Wang Z Cai L. EZH2-miRNA Positive Feedback Promotes Tumor Growth in Ovarian Cancer. Front Oncol (2021) 10:608393. doi: 10.3389/fonc.2020.608393 33718109 PMC7947696

[61] Gu YY Lu FH Huang XR Zhang L Mao W Yu XQ. Non-Coding RNAs as Biomarkers and Therapeutic Targets for Diabetic Kidney Disease. Front Pharmacol (2021) 11:583528. doi: 10.3389/fphar.2020.583528 33574750 PMC7870688

[62] Hu C Liu X Zeng Y Liu J Wu F. DNA Methyltransferase Inhibitors Combination Therapy for the Treatment of Solid Tumor: Mechanism and Clinical Application. Clin Epigenet (2021) 13(1):166. doi: 10.1186/s13148-021-01154-x PMC8394595 34452630

[63] Fernandez A O’Leary C O’Byrne KJ Burgess J Richard DJ Suraweera A. Epigenetic Mechanisms in DNA Double Strand Break Repair: A Clinical Review. Front Mol Biosci (2021) 8:685440. doi: 10.3389/fmolb.2021.685440 34307454 PMC8292790

[64] McElhinney JMWR Hasan A Sajini AA. The Epitranscriptome Landscape of Small Noncoding RNAs in Stem Cells. Stem Cells (2020) 38(10):1216–28. doi: 10.1002/stem.3233 PMC7586957 32598085

[65] Kausar S Abbas MN Cui H. A Review on the DNA Methyltransferase Family of Insects: Aspect and Prospects. Int J Biol Macromol (2021) 186:289–302. doi: 10.1016/j.ijbiomac.2021.06.205 34237376

[66] Adampourezare M Dehghan G Hasanzadeh M Hosseinpoure Feizi MA. Application of Lateral Flow and Microfluidic Bio-Assay and Biosensing Towards Identification of DNA-Methylation and Cancer Detection: Recent Progress and Challenges in Biomedicine. BioMed Pharmacother (2021) 141:111845. doi: 10.1016/j.biopha.2021.111845 34175816

[67] Wu A Tang J Guo Z Dai Y Nie J Hu W. Long Non-Coding RNA CRYBG3 Promotes Lung Cancer Metastasis *via* Activating the Eef1a1/MDM2/MTBP Axis. Int J Mol Sci (2021) 22(6):3211. doi: 10.3390/ijms22063211 33809929 PMC8048704

[68] Hu W Pei W Zhu L Nie J Pei H Zhang J. Microarray Profiling of TGF-β1-Induced Long Non-Coding RNA Expression Patterns in Human Lung Bronchial Epithelial BEAS-2b Cells. Cell Physiol Biochem (2018) 50(6):2071–85. doi: 10.1159/000495052 30423581

[69] Qin F Cao H Feng C Zhu T Zhu B Zhang J. Microarray Profiling of LncRNA Expression in the Testis of Pubertal Mice Following Morning and Evening Exposure to 1800 MHz Radiofrequency Fields. Chronobiol Int (2021) 8:1–16. doi: 10.1080/07420528.2021.1962902 34369206

[70] Wang C Ding S Sun B Shen L Xiao L Han Z. Hsa-miR-4271 Downregulates the Expression of Constitutive Androstane Receptor and Enhances *In Vivo* the Sensitivity of non-Small Cell Lung Cancer to Gefitinib. Pharmacol Res (2020) 161:105110. doi: 10.1016/j.phrs.2020.105110 32755614

[71] Puzovic V Jakic-Razumovic J. Expression of E26 Transformation Specific-1 (ETS-1) in Tumour-Infiltrating Lymphocytes (TILs) is Adverse Prognostic Factor in Invasive Breast Cancer. Breast Dis (2021) 40(1):25–31. doi: 10.3233/BD-200449 33459689

[72] Rumianek AN Greaves DR. How Have Leukocyte *In Vitro* Chemotaxis Assays Shaped Our Ideas About Macrophage Migration? Biol (Basel) (2020) 9(12):439. doi: 10.3390/biology9120439 PMC7761587 33276594

[73] Zhao G Wang Q Li S Wang X. Resistance to Hypomethylating Agents in Myelodysplastic Syndrome and Acute Myeloid Leukemia From Clinical Data and Molecular Mechanism. Front Oncol (2021) 11:706030. doi: 10.3389/fonc.2021.706030 34650913 PMC8505973

[74] Ramos Perez J Montalban-Bravo G. Emerging Drugs for the Treatment of Chronic Myelomonocytic Leukemia. Expert Opin Emerg Drugs (2020) 25(4):515–29. doi: 10.1080/14728214.2020.1854224 33280448

[75] Mahboobifard F Dargahi L Jorjani M Ramezani Tehrani F Pourgholami MH. The Role of Erα36 in Cell Type-Specific Functions of Estrogen and Cancer Development. Pharmacol Res (2021) 163:105307. doi: 10.1016/j.phrs.2020.105307 33246174

[76] Swaminathan M Wang ES. Novel Therapies for AML: A Round-Up for Clinicians. Expert Rev Clin Pharmacol (2020) 13(12):1389–400. doi: 10.1080/17512433.2020.1850255 33412978

[77] Nguyen P Safdar J Mohamed A Soubani A. Azacitidine-Induced Pneumonitis and Literature Review. BMJ Case Rep (2020) 13(10):e236349. doi: 10.1136/bcr-2020-236349 PMC7597485 33122228

[78] Samhouri Y Ursu S Dutton N Tanvi V Fazal S. Tagraxofusp Followed by Combined Azacitidine and Venetoclax in Blastic Plasmacytoid Dendritic Cell Neoplasm: A Case Report and Literature Review. J Oncol Pharm Pract (2021) 27(4):990–5. doi: 10.1177/1078155220951850 32847479

[79] Crimi E Benincasa G Cirri S Mutesi R Faenza M Napoli C. Clinical Epigenetics and Multidrug-Resistant Bacterial Infections: Host Remodelling in Critical Illness. Epigenetics (2020) 15(10):1021–34. doi: 10.1080/15592294.2020.1748918 PMC7518673 32290755

[80] Spartalis E Kotrotsios K Chrysikos D Spartalis M Paschou SA Schizas D. Histone Deacetylase Inhibitors and Papillary Thyroid Cancer. Curr Pharm Des (2021) 27(18):2199–208. doi: 10.2174/1381612826666201211112234 33308111

[81] Shetty MG Pai P Deaver RE Satyamoorthy K Babitha KS. Histone Deacetylase 2 Selective Inhibitors: A Versatile Therapeutic Strategy as Next Generation Drug Target in Cancer Therapy. Pharmacol Res (2021) 170:105695. doi: 10.1016/j.phrs.2021.105695 34082029

