# Supplementary figures and images for: Hypermethylation of the Promoter of miR-338-5p Mediates Aberrant Expression of ETS-1 and Is Correlated With Disease Severity Of Astrocytoma Patients
Source: Front Oncol. 2021 Nov 11;11:773644. doi: 10.3389/fonc.2021.773644 (PMC8632532; doi:10.3389/fonc.2021.773644)

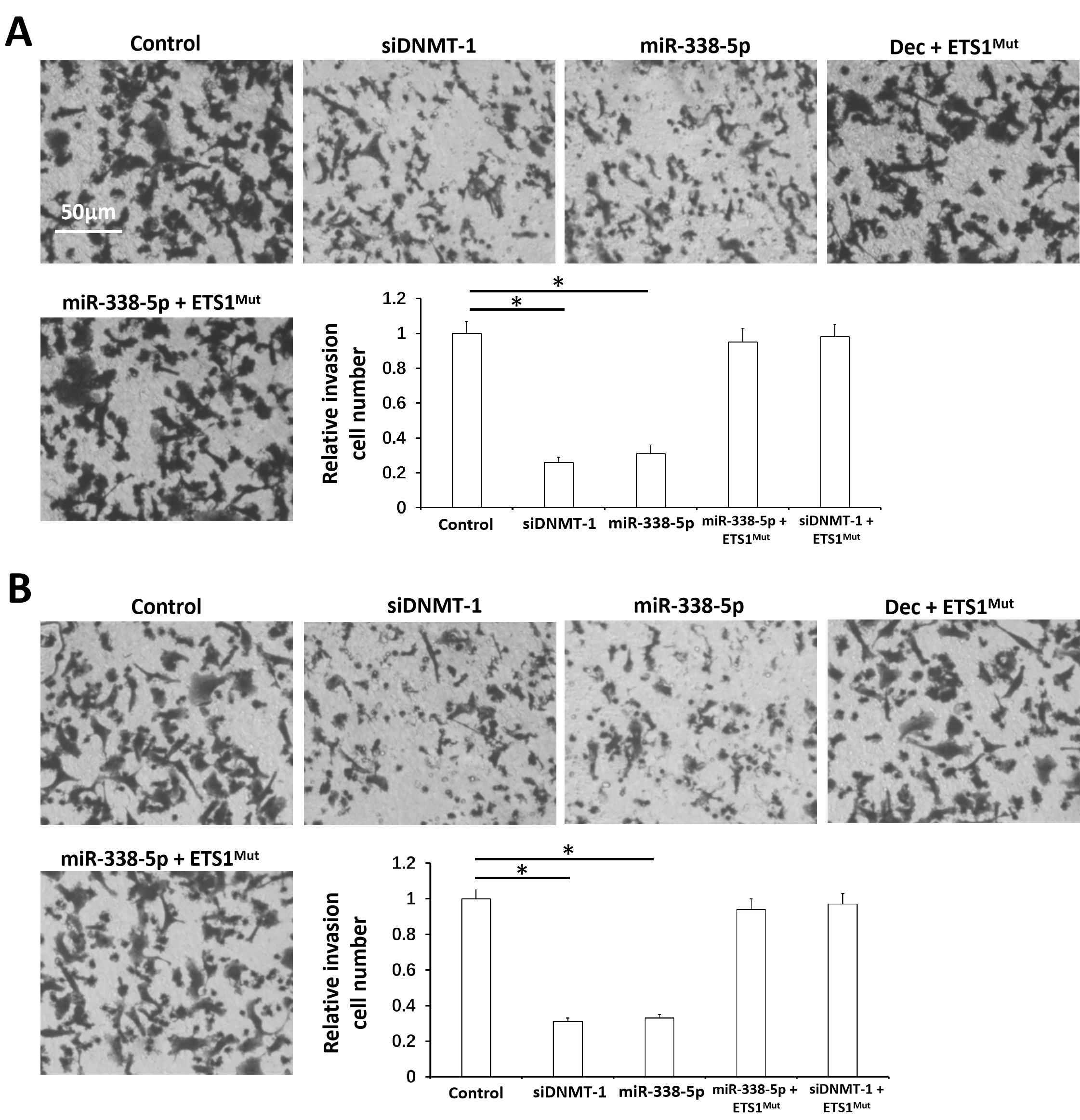

Supplement: Supplementary Figure 1 — Effects of DNMT-1/miR-338-5p on invasion of U87 and U251 cells in vitro. U87 (A) and U251 (B) cells were transfected with vectors. Then, cells were harvested for in vitro transwell experiments. Images of the invaded cells are shown, as well as quantitative results. The statistical analysis was performed by student T-test without two-tails. *P < 0.05. [file Image_1.jpg]

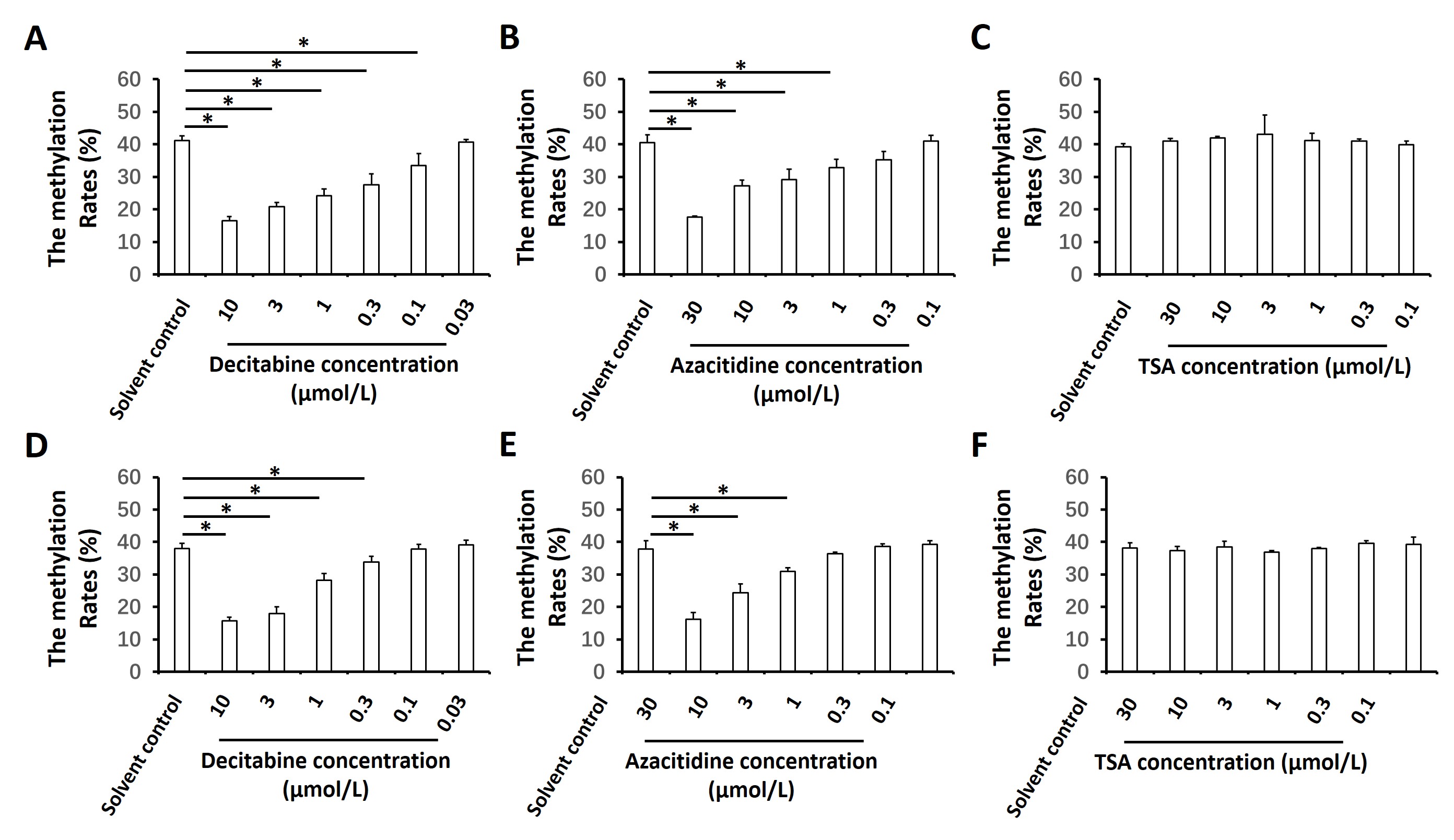

Supplement: Supplementary Figure 2 — The effect on inhibitors on the methylation rates of miR-338-5p promoter in U87 and U251 cells. The U87 (A-C) and U251 cells (D-F) cells were treated with the indicated concentrations of Decitabine (A, D), Azacitidine (B, E) or TSA (C, F), was harvested for the BSP-NGS. The results were shown as histogram of miR-338-5p promoter’s methylation rates (mean ± SD). The statistical analysis was performed by student T-test without two-tails. P < 0.05. [file Image_2.jpg]
